# Supplementary material for: An Early SARS-CoV-2 Omicron Outbreak in a Dormitory in Saint Petersburg, Russia
Source: Viruses. 2023 Jun 22;15(7):1415. doi: 10.3390/v15071415 (PMC10385080; doi:10.3390/v15071415)
Supplement: Supplementary file 1 [file viruses-15-01415-s001.zip › viruses-2451394-supplementary.pdf]

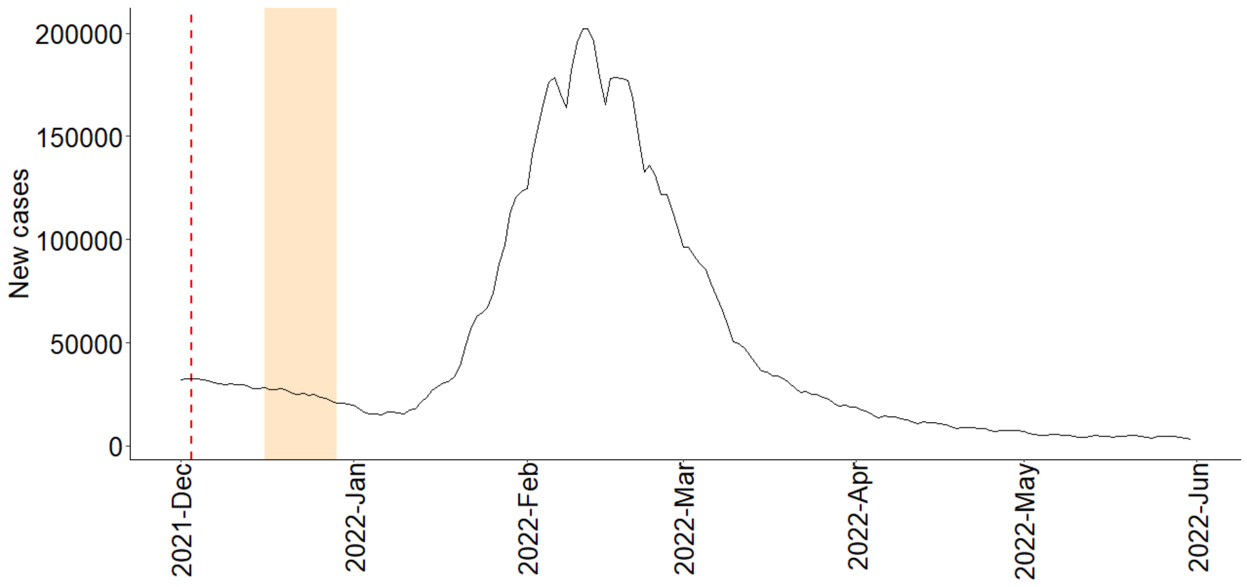

Figure S1. The daily dynamic of new cases in Russia from December 2021 to June 2022. Yellow background indicates the period of sampling in the dormitory. Dashed red line marks the time of first detection of Omicron in Russia.

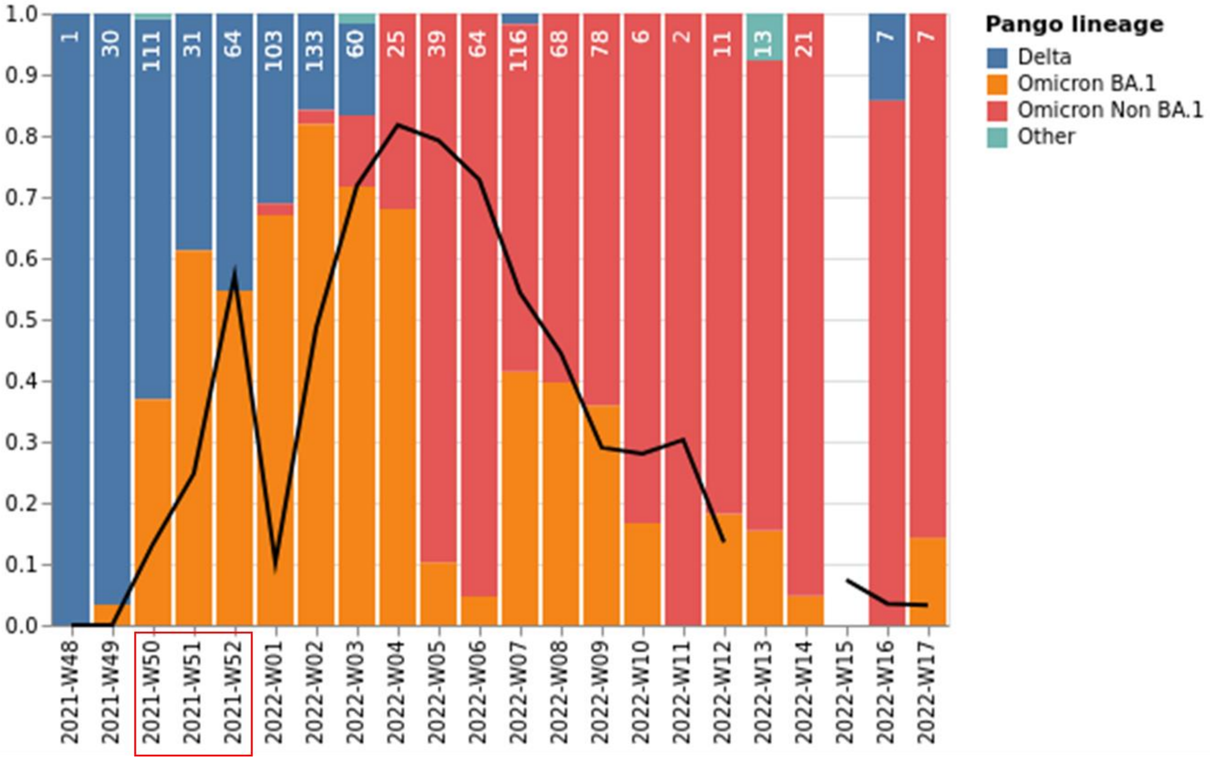

Figure S2. The fraction of Omicron among screened by PCR or among sequenced samples in Saint-Petersburg from the end of November 2021 to the end of April 2022. The numbers above the columns are the number of sequenced genomes. Colors of columns are fractions of PANGO lineages in sequenced samples.The black line is the fraction of BA.1 line according to PCR data on ins214EPE. Red rectangle marks the time of sampling of the dormitory outbreak. Dormitory samples are excluded from the plot.

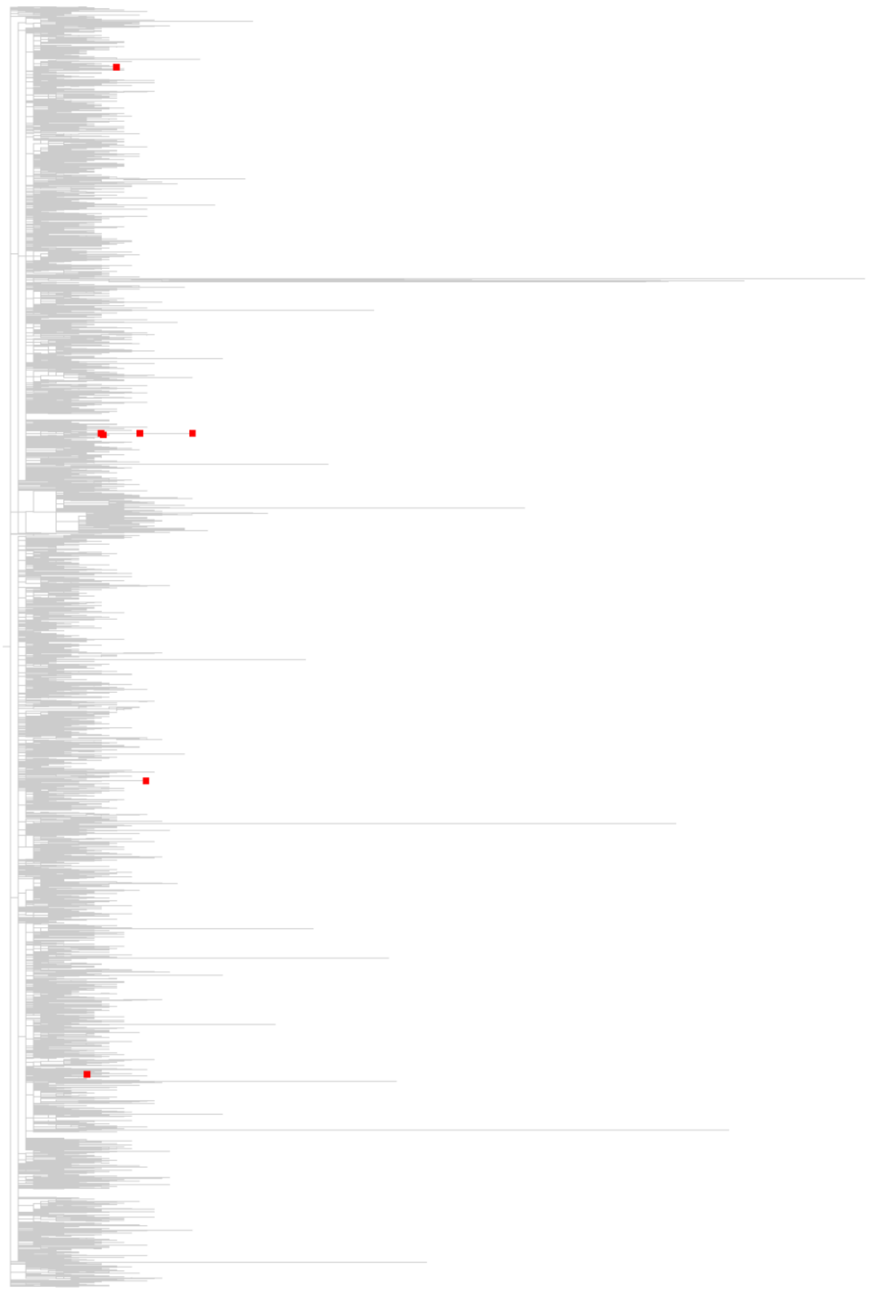

Figure S3. Dormitory samples (red) on the UShER phylogenetic tree of AY.122 obtained on 21-01-2022. All Russian samples and 1% of Non-Russian samples are shown.

Supplementary Table S1. Phylodynamic parameters for the dormitory outbreak inferred for three values of clock rate under birth-death skyline model.

Clock rate	7.25E-4<br>[6.29E-4, 8.19E-4]	9.16E-4<br>[8.20E-4, 1.01E-3]	11.1E-4<br>[10.16E-4, 12.09E-4]
Likelihood	-40833.24	-40835.25	-40836.99
Prior	782.24	798.83	811.07
TMRCA	1 December<br>[22 November,<br>9 December]	4 December<br>[26 November,<br>11 December]	6 December<br>[1 December<br>11 December]
Re1	3.79<br>[2.22, 5.67]	4.47<br>[2.48, 6.85]	5.08<br>[2.77, 7.86]
Re2	1.67<br>[0.99, 2.39]	1.80<br>[1.10, 2.55]	1.94<br>[1.20, 2.72]
Re3	2.61<br>[0.66, 5.18]	2.61<br>[0.65, 5.17]	2.61<br>[0.64, 5.14]
Sp1	0	0	0
Sp2	0.90<br>[0.71, 1]	0.91<br>[0.75, 1]	0.92<br>[0.78, 1]
Sp3	0	0	0
Sp4	0.27<br>[0.02, 0.60]	0.27<br>[0.01, 0.58]	0.26<br>[0.01, 0.58]

Supplementary Table S2. Fraction of descendants of dormitory clade among all Russian BA.1.1 samples obtained after 16th December 2021 that were present on the phylogenetic tree provided by UShER on 25th May 2022. *95% Wilson CI

Region	All Samples	Dorm-derived	Dorm-derived	Lower Bound	Upper Bound

		Samples	Fraction		
LEN	4	2	0.5	0.15	0.85
SPE	100	41	0.41	0.32	0.51
LIP	39	5	0.13	0.06	0.27
KLU	36	3	0.08	0.03	0.22
SAR	33	2	0.06	0.02	0.2
RYA	19	1	0.05	0	0.25
SMO	30	1	0.03	0	0.17
IVA	18	0	0	0	0.18
TUL	21	0	0	0	0.15
YAN	3	0	0	0	0.56
MOW	81	0	0	0	0.05
MO	4	0	0	0	0.49
PRI	33	0	0	0	0.1
ME	4	0	0	0	0.49
KL	18	0	0	0	0.18
TVE	15	0	0	0	0.2
MAG	7	0	0	0	0.35
VLA	2	0	0	0	0.66
KAM	1	0	0	0	0.95
ULY	13	0	0	0	0.23
KB	3	0	0	0	0.56
OMS	6	0	0	0	0.39
KDA	1	0	0	0	0.95
MUR	1	0	0	0	0.95

Supplementary Table S3. Fraction of descendants of dormitory clade among all non-russian BA.1.1 samples obtained after 16th December 2021. Only countries with non-zero fraction are shown.*95% Wilson CI

Country	All Samples	Dorm-derived Samples	Dorm-derived Fraction	Lower Bound	Upper Bound
Estonia	328	6	0.018	0.01	0.04
Finland	2340	11	0.005	0	0.01
Austria	1358	2	0.001	0	0.01
Israel	7087	10	0.001	0	0
Argentina	729	1	0.001	0	0.01
Denmark	2964	4	0.001	0	0
Germany	37666	40	0.001	0	0
France	10414	8	0.0008	0	0
Slovakia	2760	2	0.0007	0	0
Norway	1390	1	0.0007	0	0
India	1517	1	0.0007	0	0
Switzerland	4900	2	0.0004	0	0
Slovenia	6263	2	0.0003	0	0
Italy	3170	1	0.0003	0	0
Netherlands	4448	1	0.0002	0	0
Japan	9015	2	0.0002	0	0
Belgium	4707	1	0.0002	0	0
England	96314	18	0.0002	0	0
Scotland	21704	4	0.0002	0	0
USA	271041	1	0.000007	0	0
